# The defining pathology of the new clinical and histopathologic entity *ACTA2*-related cerebrovascular disease

**DOI:** 10.1186/s40478-015-0262-7

**Published:** 2015-12-04

**Authors:** Maria-Magdalena Georgescu, Marco da Cunha Pinho, Timothy E. Richardson, Jose Torrealba, L. Maximilian Buja, Dianna M. Milewicz, Jack M. Raisanen, Dennis K. Burns

**Affiliations:** Department of Pathology, The University of Texas Southwestern Medical Center, 5323 Harry Hines Blvd., Dallas, TX 75390 USA; Department of Radiology, The University of Texas Southwestern Medical Center, 5323 Harry Hines Blvd., Dallas, TX 75390 USA; Department of Pathology and Laboratory Medicine, The University of Texas Health Science Center at Houston, Houston, TX 77030 USA; Department of Internal Medicine, The University of Texas Health Science Center at Houston, Houston, TX 77030 USA

**Keywords:** Cerebrovascular disease, Smooth muscle actin, *ACTA2*, Mutations, ARCD

## Abstract

**Introduction:**

Smooth muscle cell contraction is an essential function of arteries and relies on the integrity of the actin-myosin apparatus. The tissue-specific α2-smooth muscle actin, encoded by *ACTA2*, is predominantly expressed in vascular smooth muscle cells. *ACTA2* mutations predispose to development of aortic aneurysms and early onset coronary and cerebrovascular disease. Based on arteriographic findings, a distinct cerebrovascular disease has been proposed for *ACTA2* heterozygous patients carrying the R179H mutation.

**Results:**

We present the first integrated analysis of a severely compromised patient with the R179H mutation and define the arterial pathology of *ACTA2*-related cerebrovascular disease. Histologically, striking morphological abnormalities were present in cerebral arteries of all sizes. Massive intimal smooth muscle cell proliferation, fragmentation of the elastic laminae and medial fibromuscular proliferation characterized large arteries whereas prominent vessel wall thickening, fibrosis and smooth muscle cell proliferation were unique changes in small arteries. The medial fibrosis and smooth muscle cell proliferation explain the characteristic radiologic appearance of “straight arteries” and suggest impaired function of mutant smooth muscle cells. Actin three-dimensional molecular modeling revealed critical positioning of R179 at the interface between the two strands of filamentous actin and destabilization of inter-strand bundling by the R179H mutation, explaining the severe associated phenotype.

**Conclusions:**

In conclusion, these characteristic clinical and pathologic findings confirm *ACTA2*-related cerebrovascular disease as a new cerebrovascular disorder for which new therapeutic strategies need to be designed.

**Electronic supplementary material:**

The online version of this article (doi:10.1186/s40478-015-0262-7) contains supplementary material, which is available to authorized users.

## Introduction

Smooth muscle cells (SMCs) form the muscle layers of tubular viscera and blood vessels [1]. Their contractile activity, generated by the actin-myosin complex, provides blood vessels and tubular viscera with motility, tonus and compliance. Unlike skeletal muscle fibers, in which the actin-myosin apparatus is organized into sarcomeres, the contractile filaments in SMC crisscross obliquely through the cell forming a lattice-like network [2]. In addition to serving a contractile function, SMCs synthesize extracellular matrix (ECM) components such as collagen, elastin and proteoglycans, similar to fibroblasts [3].

Actin is the most abundant protein in many cell types. It exists as a 42-kDa globular (G)-actin monomer and, upon poymerization, as a double-stranded helical filamentous (F)-actin structure. Six isoforms of actin encoded by different genes are present in humans, of which two α isoforms are selectively expressed in skeletal and cardiac muscles, and two cytoplasmic isoforms, β and γ, are ubiquitously expressed in muscle and non-muscle cells [4]. Two isoforms of smooth muscle actin (SMA), α2-SMA and γ-SMA, are present in SMCs in various proportions depending on the organ. Whereas γ-SMA is preferentially expressed in viscera, α2-SMA is highly expressed in vascular SMCs. Interestingly, veins but not arteries, express high levels of γ-SMA in addition to α2-SMA [5]. α2-SMA is encoded by *ACTA2* on chromosome 10q23.31.

Heterozygous *ACTA2* mutations predispose to thoracic aortic aneurysm disease (TAAD) [6]. Mutations can occur de novo and the disease has an autosomal dominant mode of transmission. A subset of *ACTA2* mutations also cause early onset coronary artery disease (CAD) and stroke [7]. The stroke phenotype has been previously described as a form of moyamoya disease (MMD) [7], but recent studies showing lack of susceptibility for MMD on chromosome 10q23 [8, 9] and a different radiologic appearance of the cerebral blood vessels suggest that a distinct cerebrovascular disease is associated with *ACTA2* mutations [10]. Among the *ACTA2* mutations, the one resulting in the R179H change in α2-SMA confers a particularly severe cerebral arteriopathy that differs from classical MMD [10, 11]. Other *ACTA2* mutations predisposing to stroke have been reported, such as those resulting in R258C/H and R39H changes [7].

In this study, we performed an integrated clinical, radiologic and pathologic analysis of a unique case harboring the R179H mutation, extending and completing previously reported analyses [10, 11]. Structural modeling of R179H and other *ACTA2* mutations involved in the stroke syndrome showed a common positioning on the actin inter-strand surface responsible for F-actin double strand bundling, providing a molecular basis for the new *ACTA2*-related cerebrovascular disease (ARCD) entity.

## Material and methods

### Autopsy, histology and digital analysis

These studies were performed in compliance with the ethical guidelines of the Helsinki Declaration and approved by the University of Texas Southwestern Medical Center and Parkland Hospital Health Center ethical committees for research on human subjects. The genetic identification, brief clinical history and radiologic appearance of the patient carrying the heterozygous *ACTA2* mutation resulting in the R179H change was described in the addendum [12] and as patient 6 [10], 4 years before she expired. Her autopsy and that of a gender, race and age-matched control patient succumbing of cirrhosis were performed in accordance to the UT Southwestern/Parkland Hospital regulations. These patients were of normal weight and comparable height and were free of other risk factors for cardiovascular disease, such as smoking, diabetes, hypercholesterolemia, hypertension or obesity. Representative sections were obtained from all the organs, including aorta. Brains were fixed for 2 weeks in formalin and the following cerebral arteries were carefully dissected prior to sectioning: supraclinoid internal carotid arteries (ICAs), middle cerebral arteries (MCAs), anterior cerebral arteries (ACAs), posterior communicating arteries (PComs), posterior cerebral arteries (PCAs), basilar artery, vertebral arteries (VAs), superior cerebellar arteries and posterior inferior cerebellar arteries. Three 2-mm long fragments were obtained when possible for each artery. Paraffin-embedded sections were processed for hematoxylin-eosin (H&E), Masson trichrome and Verhoeff van Gieson elastic stains for all the arteries. Immunohistochemistry (IHC) was performed on selected sections with α-SMA antibody (clone 1A4, pre-diluted, Ventana Medical Systems, Tucson, AZ). Images were acquired at various magnifications with an Aperio Scanscope CS2 whole slide image system (Leica Biosystems, San Diego, CA) and the measurements of thickness or diameter were performed by using ImageScope software, version 12.1.0.5029 on images at 20x magnification. Measurements of large artery intima and media thickness were performed on the H&E sections of the arterial fragment showing the thickest intima, at maximum and mean thickness, respectively. Measurements of the small vessel wall were performed at mean thickness and caution was taken when vessels were not circular. The measurements of small vessel lumen diameter were performed on α-SMA labeled sections and, when the lumen was elliptic rather than circular, the formula (D + d)/2 was used, where D is the long axis and d, the small axis of the ellipse. SMC nuclei were counted in random fields of large artery media, in a range of 130–361 nuclei/field, and normalized to area, by using the analysis tools in Adobe Photoshop CS6, version 13.0 (Adobe Systems Inc., San Jose, CA).

### Radiologic imaging and analysis

Cross sectional imaging studies, including computed tomography (CT) and magnetic resonance imaging (MRI) performed as part of routine clinical care and available from the hospital picture archiving and communication system (PACS), were reviewed by a neuroradiologist. Imaging findings were compared to published literature regarding *ACTA2* mutations and MMD. Measurements of luminal diameters and cross sectional areas of the main intracranial arteries were performed on source images of a CT angiography study of the patient, as well as of an age and gender matched second normal control different from the autopsy control, by using the Osirix open source DICOM viewer [13]. Multiplanar reformats of isotropic CT data were performed to obtain long and short axis views of the arteries of interest. Linear measurements were obtained for luminal diameter in the mid portion of the vessels on long axis views and region of interest circular measurements were obtained for area measurements in the short axis views.

### Statistical analysis

**Fig. 1:**
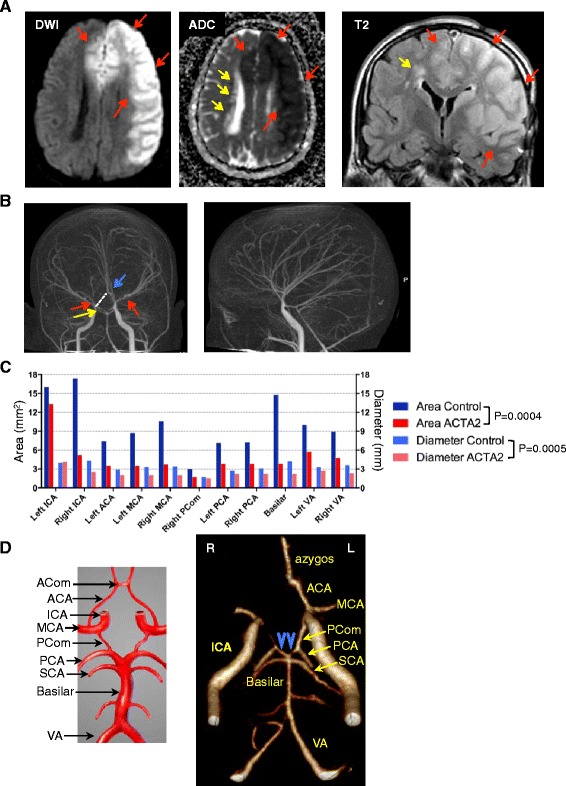
Radiologic characteristics of ARCD. **a**. MRI axial diffusion weighted image (DWI) and corresponding apparent diffusion coefficient (ADC) map, and coronal T2W-FLAIR image showing acute infarct with restricted diffusion involving the territories of the left MCA and bilateral ACAs (red arrows). Chronic periventricular leukoencephalopathy (yellow arrows) appears with hyperintensity on FLAIR images and increased diffusivity on ADC map. **b**. CT angiography demonstrating the straight course of most of the intracranial arteries, as well as lack of basal collaterals, which are typically seen in MMD. Narrowing of the supraclinoid segment of right ICA (yellow arrow) and bilateral MCAs (red arrows), and the azygos configuration of the A2 segment of ACA (blue arrow) are also shown. The missing right ACA A1 segment is indicated by a punctuated line and explains involvement of bilateral ACA territories with a left ICA occlusion. **c**. In vivo lumen diameter and area measurements by CT angiography (see also Additional file 1: Figure S1). The arterial measurements are compared to a normal age and gender-matched control. No left posterior communicans artery (PCom) was identified for the control. Control and mutant datasets are compared by two-way ANOVA. **d**. The configuration of the circle of Willis in the ARCD patient shows a combination of anatomic variants in both the anterior – lack of A1 of right ACA and common anterior azygos trunk- and posterior parts - “fetal-type” variant of PCAs with hypoplastic P1 segments (blue arrowheads). A drawing depicting the circle of Willis is shown for comparison

**Fig. 2:**
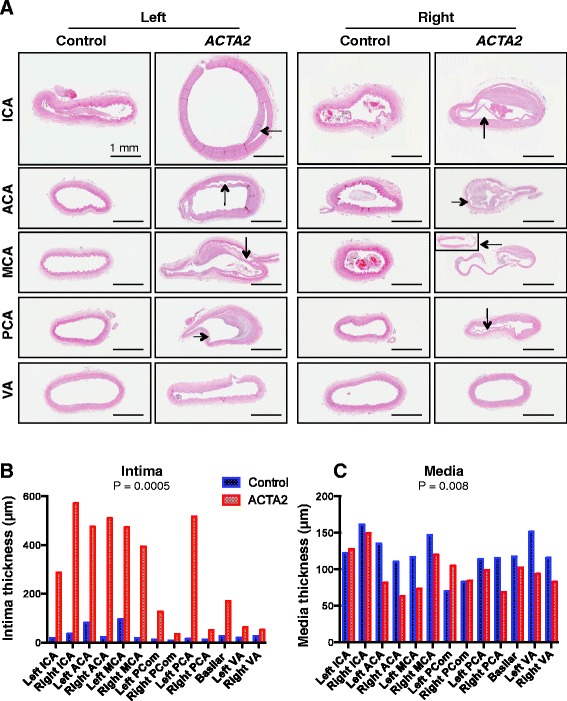
Analysis of large cerebral arteries. **a.** H&E of major branches of the circle of Willis and vertebral arteries (VA). Note marked splitting between the intima and media in the *ACTA2* mutant (arrows), resulting in complete separation of the tunics in the right MCA (insert shows media). **b-c.** The thickness of intima (**b**) and of media (**c**) were measured at maximum and mean layer thickness, respectively. Control and mutant datasets are compared by two-way ANOVA

Numerical data were examined for normality of distribution and expressed as mean ± SEM by using the GraphPad Prism program (GraphPad Software, La Jolla, CA). The two-way ANOVA test in GraphPad Prism was used for comparison across the control and mutant large vessels series in Figs. 1c, b and 2c and the two-tailed *t*-test with Welch’s correction for variances significantly different was used to analyze the differences between groups for the rest of the analyses. Statistical significance was considered for *P*< 0.05. Graphs were plotted with GraphPad Prism program.

### 3D modeling

The various α2-actin mutations were mapped and aligned in the monomeric α1-actin 3D structure (Protein Data Base accession number 1J6Z). The models of wild-type or mutant α2-actin residues were generated by using PyMol (Version 1.5.0.4 Schrodinger, LLC), as previously described [14].

## Results

### The characteristic clinical and angiographic features of ARCD

A 31-year-old female was brought unresponsive to the emergency room by her husband. Upon arrival at an outside hospital, the patient was nonverbal, not following commands and without purposeful movements. There was suspicion of seizure activity due to face and finger twitching, and the patient was intubated for airway protection. The patient had a complex prior medical history including multiple previously repaired aneurysms (aortic root, arch and right axillary artery at age 25 years, and thoracoabdominal aorta at age 26 years), surgical closure for a persistent ductus arteriosus in childhood, congenital mydriasis, intestinal malrotation diagnosed at laparotomy at age 2 years, left salpingo-oophorectomy, colecystectomy at age 23, and obstructive and restrictive chronic lung disease. The patient also had a history of left superficial temporal artery encephalodural synangiosis 2 years and 9 months prior, due to diagnosed MMD, associated with body paresthesias and transient weakness. The constellation of vascular abnormalitites that include aortic aneurysms and persistence of patent ductus arteriosus, with involvement of SMCs in other organs leading to congenital absence of the iris, malrotation of the gut and primary pulmonary hypertention was previously described in individuals with *ACTA2* mutations resulting in a change at R179 and was classified as multisystemic smooth muscle dysfunction syndrome [12]. The brief clinical and radiologic description of this patient carrying the R179H mutation was included in the addendum of the latter study [12] and was also mentioned in the table (patient 6) of the study by Munot et al. [10]. However, her brain imaging findings are comprehensively described below, in this study. A brain MRI at the time of admission revealed large left MCA and bilateral ACA infarcts (Fig. 1a, red arrows), with additional evidence of chronic small vessel white matter disease (periventricular hyperintensities; Fig. 1a, yellow arrow) and postsurgical findings related to prior left pterional craniotomy for revascularization. Head CT confirmed a large hypodense area of acute infarction involving the left MCA and bilateral ACA territories. The patient was admitted to the Neurology intensive care unit for supportive care. At arrival, the patient was comatose with Glasgow Coma Score of 7/15. An electroencephalogram revealed no evidence of seizure activity. Suppression of background activities within the left frontocentral region was observed, corresponding to the patient’s ischemic stroke. Follow-up head CTs at one and two days after arrival did not demonstrate evidence of hemorrhagic transformation, but showed progression of ischemic injury in the posterior left MCA territory, worsening edema, mass effect and rightward midline shift. The family deferred surgical decompression according to the patient’s previously stated requests. The patient expired later on the same day and a postmortem examination was requested.

Review of patient’s prior neuroimaging studies demonstrated findings consistent with her history of *ACTA2* mutation-related vasculopathy. Importantly, the CT angiography demonstrated an unusual straight course of most of the intracranial arteries and an absence of the characteristic collaterals seen in MMD (Fig. 1b). To examine the capacity of the large cerebral vessels, we performed measurements on CT angiography images of the luminal cross sectional area and diameter (Additional file 1: Figure S1). Both parameters were significantly lower in the *ACTA2*-mutant patient compared to a normal age- and gender-matched control (Fig. 1c), suggesting chronic hypoperfusion of the brain. Exceptions were noted in the sites of increased compensatory flow, such as for the left ICA that supplied the right ACA territory through the azygos ACA in addition to the normal left ACA territory, and for the posterior communicating arteries, that supplied the main flow to PCAs due to hypoplasia of the P1 segments of the PCAs (Fig. 1d).

### Marked abnormalities of intima and media in *ACTA2* mutant large-size cerebral arteries

Gross examination of the brain showed mildly increased weight (1300 g) with signs of edema of the left cerebral hemisphere: widening and flattening of the gyri, softening of the parenchyma, left lateral ventricle narrowing with mild midline shift, mild left subfalcine herniation and left uncal herniation (Additional file 1: Figure S2). Colpocephaly-like dilatation of the trigone and occipital horns of the lateral ventricles, was also noted (Additional file 1: Figure S2, red arrow). The microscopic examination of representative sections confirmed the radiological findings of infarct and showed generalized acute neuronal and glial necrosis, mild neutrophilic reaction and endothelial swelling in the left MCA, ACA and right ACA territories (Additional file 1: Figure S3). Occasional acute ischemic neuronal necrosis was also seen in the right basal ganglia and bilateral thalami (not shown), suggesting global ischemia.

Gross examination of the major arteries of the cerebral circulation confirmed the marked narrowing of the lumen of the right supraclinoid ICA, the unilateral azygos configuration of ACAs and the hypoplasia of the P1 segments of the PCAs noted radiographically (Additional file 1: Figure S4). Although these variants in the anterior and posterior parts of the circle of Willis circulation account each for 2–10 % in the general population [15], the combination of these variants in a single individual most likely reflects a developmental abnormality. A comparative histological analysis was further carried out between the *ACTA2* patient’s arteries and those of a gender and age-matched control (Fig. 2). H&E staining revealed marked thickening of the intima in all major arteries of the anterior cerebral circulation and variable intimal thickening of the arteries of the posterior cerebral circulation (Fig. 2a-b). The intimal thickening explains the decreased lumen diameter and area observed angiographically (Fig. 1c) and the propensity to develop thrombosis. Indeed, both the left MCA and right ICA contained organizing thrombi at autopsy (not shown).

A striking splitting between the intima and the media at the level of the internal elastic lamina was observed in the *ACTA2* mutant arteries compared to arteries from the age-matched control patient, especially in the anterior circulation (Fig. 2a arrows). A minimal degree of splitting has been described as an artifact and attributed to the postmortem contraction of the media [1]. The excessive splitting present in the *ACTA2* mutant arteries is a postmortem artifact as well but suggests additional deregulation of adhesion between intima and media.

The measurement of the average thickness of the media revealed comparable medial thinning in both anterior and posterior cerebral circulations of the *ACTA2* mutant (Fig. 2c). Exceptions were noted in the cases of compensatory flow, such as for the left ICA and the two posterior communicating arteries.

**Fig. 3:**
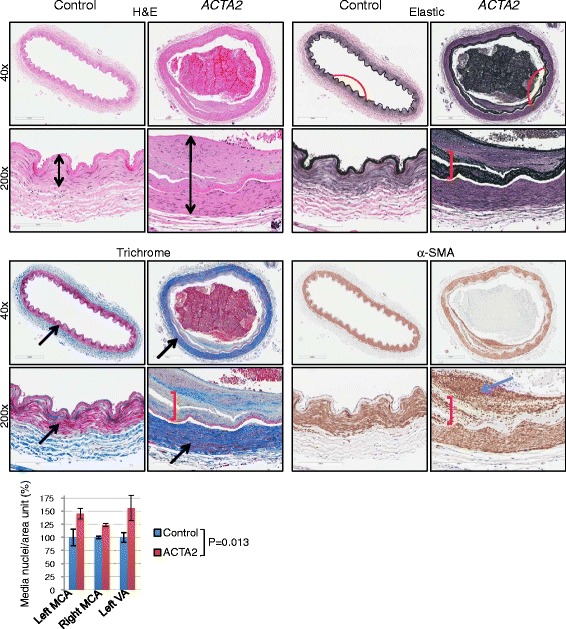
Large artery changes in ARCD. Serial sections of left MCA labeled with H&E, elastic and trichrome special stains and with α-SMA IHC show thickened arterial walls (double headed arrows), flattening (red curve), splitting and disorganization (red bracket) of the internal elastic lamina, marked fibrosis of the media (black arrows) where SMCs stain red and increased collagen stains blue with Trichrome, and SMC proliferation (blue arrow) of intima in the *ACTA2* mutant as compared to control. The numbers of SMC nuclei from the media of the indicated large cerebral arteries were counted on random fields from H&E sections, normalized to area and expressed as mean ± SD. Control and mutant groups for each artery were compared by *t*-test

Special stains for elastic fibers (Verhoeff van Gieson) and ECM (Trichrome), and IHC with α-SMA antibody for SMCs revealed severe changes present in *ACTA2* mutant large cerebral arteries (Fig. 3). Overall, the combined wall thickness of the *ACTA2* mutant arteries was significantly increased compared to normal arteries due to increased intima thickness (Fig. 3, H&E, double-headed arrows). The elastic laminae, labeled in black by the silver elastic stain, were less folded (Fig. 3, elastic stain, red arcs) and were thickened, split and fragmented (Fig. 3, elastic stain, red bracket). A modified collagen content in areas highlighted by the elastin stain was suggested in the trichrome stain (Fig. 3, trichrome stain, red bracket). An increased amount of ground substance in the thickened intima was also apparent. One of the most striking abnormalities encountered in all *ACTA2* mutant arteries was the substantial increase in collagen within the media (Fig. 3, trichrome stain, arrows). This excessive collagen production was accompanied by a mild increase in the number of SMCs in the media of large arteries (Fig. 3, graph). Together with the overall increased wall thickness, the severe media fibrosis most likely explains the characteristic, and possibly pathognomonic, straight appearance of the cerebral arteries on imaging studies (Fig. 1b).

IHC for α-SMA showed massive SMC proliferation in the intima and SMC dissection through the split internal elastic lamina (Fig. 3, α-SMA, blue arrow and red bracket, respectively).

### Unique *ACTA2* mutant phenotype of small-size arteries

**Fig. 4:**
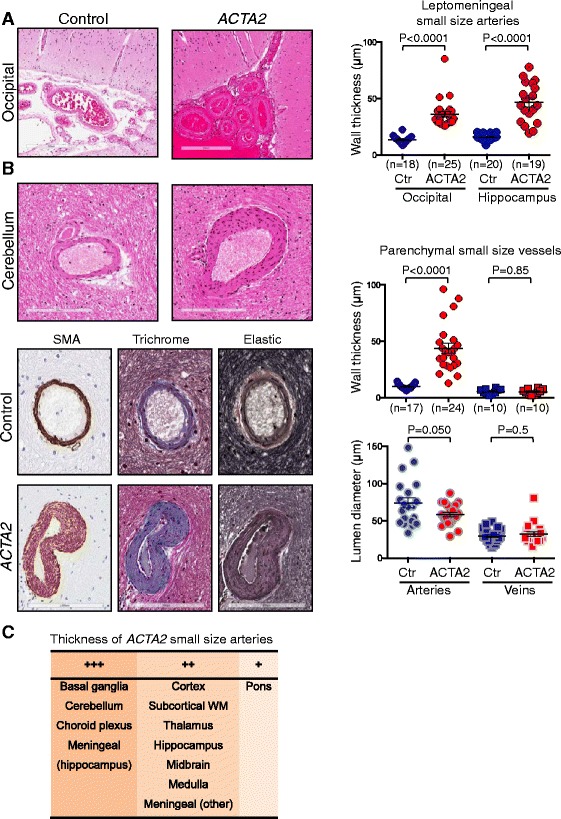
Small-size artery disease in ARCD. **a**. H&E of leptomeningeal vessels overlaying the calcarine cortex. The graph shows the distribution and mean ± SEM of the transmural thickness for leptomeningeal small arteries from two brain regions. **b**. Analysis of parenchymal small vessels from the cerebellum shows thickened walls with characteristic SMC proliferation and fibrosis. The quntifications of transmural wall thickness and lumen diameter are shown for both small arteries and veins. The scoring of lumen diameters was performed on 20 α-SMA labeled vessels in each category. Control and mutant groups for each category were compared by *t*-test. **c**. Distribution of the *ACTA2* mutant small size arteries with variable wall thickness across brain regions. +++, ++ and + represent 2.5–4, 1.5–2.5 and <1.5 fold increase vs. control

Examination of the small size arteries in various regions of the brain revealed significant abnormalities in the *ACTA2* mutant vessels (Fig. 4). Analysis of the wall thickness of leptomeningeal small size arteries in two regions of the brain, occipital and hippocampal, showed significantly thicker *ACTA2* mutant arteries compared to control (Fig. 4a, graph).

Likewise, the walls of the patient’s small intraparenchymal arteries were significantly thicker than those from the control case arteries, whereas veins showed no difference (Fig. 4b, upper graph). The sparing of the veins may reflect the presence of γ-SMA in these vessels [5]. The measurement of the lumen diameter showed a trend towards a smaller lumen in the *ACTA2* mutant, although the majority of the small arteries appeared to have lumens comparable to those in the control case. Luminal dimensions of small veins were similar in patient and control sections (Fig. 4b, lower graph). Histological analysis of the arterial walls revealed massive SMC proliferation and marked fibrosis (Fig. 4b, lower panels), similar to that noted in the media of large-size arteries. The marked proliferation of SMCs in the small size arteries appears to be a unique feature of this disease and has not been observed in other cerebral small size vessel diseases [16].

**Fig. 5:**
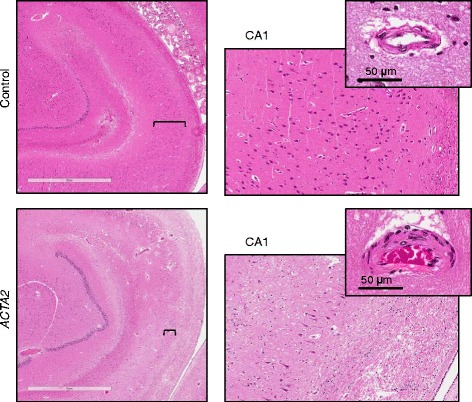
Neuronal depletion in ARCD. H&E sections of the right hippocampus show severe neuronal depletion in ARCD as compared to control, more prominent in the CA1 region. The right panels represent higher magnification fields (10x) of the CA1 region marked by brackets. Insets show small parenchymal arteries that have thickened walls in ARCD

Overall, the small arteries had thickened walls in all brain territories (Fig. 4c), with the highest thickness of 2.5-4 fold more than control in the basal ganglia, cerebellum, choroid plexus and hippocampal leptomeninges. Noteworthy, these abnormalities were also present in the subcortical white matter (Additional file 1: Figure S5). Although small infarcts or hemorrhages were not observed, radiologic signs of periventricular leukoaraiosis (Fig.1a, yellow arrow), corresponding microscopically to areas of rarefaction of the periventricular neuropil and axonal damage (Additional file 1: Figure S6), support the possibility of chronic ischemic injury. Additional evidence of chronic ischemic damage in the form of substantial neuronal loss was noted in the hippocampus, particularly in the CA1 region (Fig. 5). This generalized chronic arteriopathy might be responsible for cognitive changes, such as those observed in other hereditary cerebral small vessel diseases [16], and this aspects warrants further in depth clinical investigation.

### Systemic manifestations in ARCD: broad involvement of arterial SMCs

To assess and compare the extent of the vascular disease in other regions beside the brain, we examined representative sections of various organs. The aorta showed thinning of the wall formed by the intima and media (Additional file 1: Figure S7, double headed arrow), in contrast to cerebral arteries, for which the exuberant intimal fibromuscular proliferation accounts for increased thickness. The prominent loss and fragmentation of the elastic lamellae (Additional file 1: Figure S7 arrowheads and elastic stain) replaced by disorganized ECM and SMCs explains the decreased resistance to blood pressure and the formation of aneurysms. The small arteries of the aortic vasa vasorum had thickened walls (Additional file 1: Figure S7, arrows), similar to the small arteries in the brain. Similar histopathologic findings of the aorta have been previously described in patients with the R179H as well as other *ACTA2* mutations, including that resulting in the R258H change [6, 12].

The large size muscular arteries in various organs, including those from the kidney, liver and branches of the aorta, showed intimal fibromuscular proliferation, although this change was not as extensive as that seen in the large size cerebral arteries of the anterior circulation (Additional file 1: Figure S8A, D). The small size arteries in the connective tissue and parenchyma of various organs had thicker walls, similarly to those observed in the brain, indicating that this is a generalized defect induced by the *ACTA2* mutation (Additional file 1: Figure S8A-D). The medial thickening of the myocardial arteries (Additional file 1: Figure S8C) is similar to the one previously described for the subset of *ACTA2* mutations predisposing to CAD [7]. Although not as dramatic as the ischemic changes noted in the brain, evidence of ischemic organ damage was seen in the heart, manifested by areas of fibrosis and cardiomyocyte hypertrophy (Additional file 1: Figure S8C), and in the kidney, with areas of transcortical glomerulosclerosis along the interlobular artery territory. Interestingly, as previously reported [12], architectural abnormalities that could not be clearly related to a vascular dysfunction – specifically panacinar emphysema – were present.

### R179H maps to the interstrand F-actin surface and disrupts F-actin bundling

**Fig. 6:**
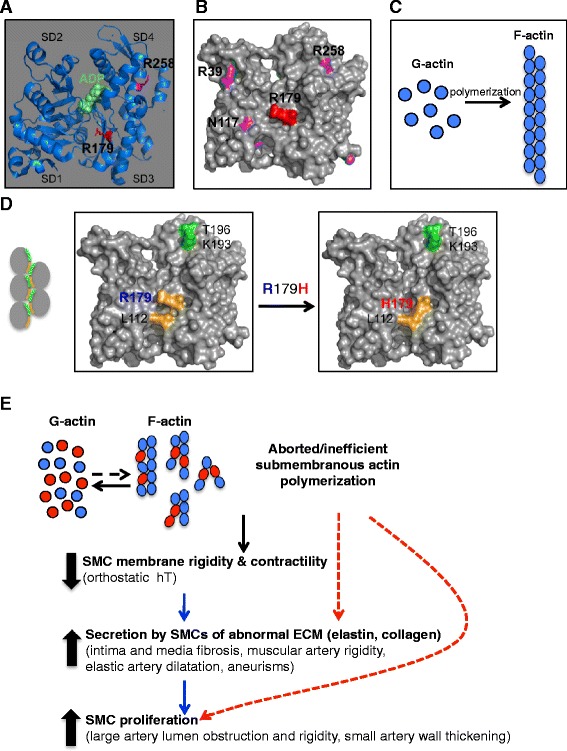
R179H 3D model and mechanism of action in ARCD. **a**. Ribbon 3D representation of monomeric α1-actin oriented to view the interstrand surface shows the 4 actin subdomains (SD), the ADP/ATP-binding site, and the positioning of R179 and R258 on the interstrand surface. **b**. Surface projection of ARCD mutations to the same surface as R179 suggests similar mechanisms of actin polymerization disruption. **c**. Diagram of monomeric G-actin polymerization into double-stranded F-actin. **d**. Surface representation shows projection of the R179 side chain to the interstrand surface (left panel). Also represented are residues involved in interstrand contact: R179 and L112 (in gold) from one actin subunit contact K193 and T196 (in green) from the paired subunit, as schematized in the cartoon. The contact interface is altered by mutation to H179 (right panel). **e**. Pathogenesis model of ARCD. F-actin polymerization from a pool of wild-type (blue) and mutant (red) monomers results in variably defective filaments depending on the mutant:wild-type monomer ratio and the relative mutant monomer positioning into the F-actin chain. Blue arrows indicate compensatory mechanisms and red dotted arrows possible intrinsic phenotypes of SMCs with mutant *ACTA2*

To dissect the role of *ACTA2* mutations in ARCD, we mapped the position of two of the most pathogenic mutations, R179H and R258H, in a 3D structure model of actin (Fig. 6a). Whereas R179 localizes to a short β-strand in actin subdomain 3, R258 is situated in an α-helix in subdomain 4. Both residues have side chains pointing to the same surface of the molecule, relatively at distance from the ADP/ATP binding site (Fig. 6a, b). The common surface is the one involved in pairing of F-actin strands [17, 18]. F-actin is a double-stranded filament resulting from the non-covalent polymerization of monomeric G-actin via intra-strand and inter-strand hydrophilic and electrostatic bonds (Fig. 6c). Both residue R179 and R258 have been reported to contribute to the inter-strand bridging [17]. In the case of R179, a contact (Fig. 6d) takes place between R179 in subdomain 3 and L112 in subdomain 1 (in gold, Fig. 6d) from one molecule and K193 and T196 in subdomain 4 (in green, Fig. 6d) of the paired molecule [17]. The modeling of the R179H change shows merging of the H179 and L112 side chain projections with significant alteration of the contact surface (Fig. 6d right panel) that very likely impinges on the inter-strand pairing.

Both R179 and R258 pathogenic mutations have been described in α2-SMA and γ2-SMA, predisposing for TAAD/stroke and megacystis-microcolon-intestinal hypoperistalsis syndrome, respectively [19]. Remarkably, the residue R179 (R177 in α1-actin) is the site of ADP-ribosylation by Clostridium botulinum, perfringens and difficile C2, iota and transferase CDT toxins, respectively [20]. ADP-ribosylation at this site results in steric hindrance of actin monomer interactions and blockage of polymerization. Mutagenesis of R179 to D in β-actin has been shown to significantly lower polymerization rate and polymer viscosity [21], suggesting a critical role in actin polymerization. While we modeled the structural effect of the R179H mutation for the first time in this report, the role of the R258H mutation that alters the same inter-strand interface as R179H (Fig. 6a-b) has been extensively studied [22] and R258H mutagenesis has been shown to cause F-actin polymerization defects [23, 24].

## Discussion

Cerebrovascular disease is a major health problem in developed countries. An understanding of the heterogeneous nature of the abnormalities manifesting clinically as cerebrovascular disease is paramount for developing disease-specific therapies. This report represents the first comprehensive histopathologic analysis of severe cerebrovascular disease resulting from an *ACTA2* mutation (ARCD), expanding our knowledge of the pathological spectrum of cerebrovascular disease. ARCD is a systemic disease that affects arteries of all sizes. The striking histopathologic changes of intima and media fibromuscular proliferation of the arteries in the anterior cerebral circulation underlie the characteristic “straight arteries” angiographic sign. While the fibromuscular proliferation of the intima noted in this patient is also a feature of atherosclerosis and MMD [25–27] several aspects distinguish the changes seen in this case from either atherosclerosis or MMD. Macrophage accumulation and cholesterol-rich plaques characteristic of atherosclerosis were not observed. Several differences with MMD were noted as well, including decreased folding of the internal elastic lamina in cross sections as opposed to more complex folding of the internal elastic lamina sometimes seen in MMD [26]. Additionally, the medial changes evident in this case - fibrosis and SMC proliferation - differ from the attenuation of the media in MMD [25, 26]. Perhaps most significantly, the compensatory small vascular collaterals characteristic of MMD were not a feature of this case, and it is possible that the altered composition of the arterial walls in ACRD may play a role in this. In MMD, the collateral vessels and most likely the revascularization following encephalodural synangiosis develop as a response to ischemia and release of angiogenic growth factors, such as vascular endothelial growth factor and others [27]. Although the same compensatory response to ischemia is probably elicited in ARCD, the growth factors produced in the ischemic tissue may not diffuse properly to the endothelial cells through the thick vasculature. Alternatively, a structural deficiency of the vessels in ARCD could interfere with the sprouting of the collateral vascular channels seen in MMD. In this respect, remodeling of the ECM and maintenance of a balance between angiogenic and anti-angiogenic factors, which are in part secreted by SMCs, is necessary for efficient angiogenensis [28]. As the ECM is clearly deregulated in ARCD arteries, most likely as a direct consequence of impaired secretion by mutant SMCs, future work to delineate these possibilities might have therapeutic implications for potential revascularization.

ARCD diffusely involved small arteries and showed histopathologic changes of fibrosis, and SMC proliferation, leading to increased wall thickness that reached in some areas 4-fold higher values than the control. These small vessel changes are unique among hereditary small vessel arteriopathies that, in contrast to ARCD, are characterized by degeneration of SMCs [16, 29]. This characteristic SMC proliferation was present systemically in the small vessels of various organs - lymph node medulla, liver, lung, heart, submucosa and serosa of esophagus, stomach and uterus - and in fibroadipose tissue but not in the sampled abdominal skin. As previously reported, the vasa vasorum in the adventitia had also increased wall thickness [6]. As these patients frequently undergo multiple surgeries, this disease may be suspected when small vessels with SMC proliferation are detected.

The generalized arteriopathy of ARCD induced secondary organ changes, the most prominent and life threatening being in the brain. These were manifested by both acute ischemic change, such as stroke, and chronic ischemic change in the form of leukoencephalopathy and neuronal loss. The MRI T2-weighted hyperintensities in the periventricular white matter (leukoaraiosis) have been previously observed in *ACTA2* patients with R179 mutations [10, 12, 30–32] but the histopathologic basis has not been clearly defined. We found areas of rarefaction containing axonal swellings at these sites, similar to lesions described in hereditary diffuse leukoencephalopathy with axonal spheroids and sometimes also seen in CADASIL [16, 33]. Moreover, the colpocephaly, a developmental abnormality that has been linked to white matter underdevelopment of various causes [34], has been also reported later in life in this patient and in a second patient carrying the R179H mutation [12]. This colpocephaly-like ventricular dilatation is suggestive of progressive chronic ischemic damage of the periventricular white matter in ARCD patients. Interestingly, the severe neuronal loss in the hippocampus, predominantly in the CA1 area, is most likely attributable to chronic ischemia as well. This finding is similar to the changes described in hippocampal sclerosis, which are considered to be a consequence of age-related angiopathy in some cases [35].

ARCD has both common and distinctive features from other vascular syndromes, the former stemming most likely from the interplay of secondary compensatory mechanisms following arterial injury. To integrate ARCD into the broader field of vascular disease, we modeled its pathogenesis starting with its characteristic structural alteration, the R179H α2-SMA mutation (Fig. 6e). Based on our structure modeling, the common mechanistic denominator for the R179H and R258H mutations described in ARCD [10] is most likely the destabilization of the F-actin inter-strand interface. Predictably, the severity of inter-strand bundling disruption depends on the mutant:wild-type ratio protein concentration and on the relative position of the mutant protein incorporated into the actin filament (Fig. 6e). These events may have secondary allosteric effects on the association of actin with actin-binding proteins, such as formin, which is required for nucleation and elongation of F-actin, thus causing severe disruption of F-actin polymerization [22]. Because of its critical positioning, the mutation of R179 might cause a more severe disruption of actin polymerization than other mutations, which may explain the more severe and generalized phenotype reported for R179 mutations [12]. How the alterations of actin polymerization translate into the morphogenetic abnormalities observed in the cases of ARCD is not known. It has been shown that only a small amount of actin in SMCs undergoes active polymerization in a submembranous network that serves to strengthen the membrane for the transmission of force generated by the contractile apparatus to the extracellular matrix [36]. An abnormal submembranous actin polymerization would give rise to decreased SMC membrane rigidity and thus decreased contractility (Fig. 6e). Surprisingly, the *ACTA2* knock-out mice share with the human mutant *ACTA2* patients only the blood pressure dysregulation due to contractility impairment. Otherwise, these mice are viable and do not have morphological cardiovascular defects, most likely due to compensatory expression of skeletal muscle actin in their vessels [37]. This suggests a dominant-negative effect of the mutant α2-SMA on vasculature morphogenesis with alteration of other SMC functions than contractility. Indeed, the fibrosis of media and accumulation of abundant ground substance and disorganized elastin in the intima of the ARCD arteries suggests that the secretory function of SMCs is deregulated. Whether SMCs with R179H mutant α2-SMA secrete abnormal ECM directly or as a consequence of arterial damage due to inefficient contractility is not clear. Due to similarities with other TAADs and cerebral vasculopathies with abnormal ECM [29, 38], a compensatory secretion of abnormal ECM is probably taking place (Fig. 6e). Both elastin knockout mice and humans with supravalvular aortic stenosis characterized by loss-of-function mutations on one elastin allele have uncontrolled SMC proliferation in the intima leading to arterial stenosis and death [3, 39]. It has been shown that the intimal SMC proliferation results from decreased deposition of insoluble elastin and, conversely, that addition of insoluble elastin inhibits SMC proliferation [39]. Strikingly, massive intimal SMC proliferation was present in most of the large size cerebral arteries in ARCD, most likely due to defective elastin deposition [39]. Although this loop may be involved in the characteristic proliferation of the SMC population from the media of large arteries or wall of small arteries, the hypothesis of an intrinsic hyperproliferative phenotype of mutant *ACTA2* SMCs remains to be explored (Fig. 6e). Notably, SMCs from *ACTA2* knock-out mice proliferate and migrate more rapidly than control SMCs [40].

## Conclusions

In conclusion, this study presents the first comprehensive analysis of cerebrovascular changes in a patient with ARCD and offers morphological explanations for many of the clinical findings. The cues that trigger the global artery morphogenetic defects are yet to be found. These may not only explain why the correction of the vascular flow by arterial bypass, a procedure successfully used in MMD, usually fails in ARCD patients, but also unravel new treatment options directed to target the arterial obstructive pathology in ARCD.

## Additional file

Additional file 1:
**Figure S1**. CT angiography measurements of luminal cross sectional area and diameter in ARCD. **Figure S2**. Gross appearance of the ARCD brain with recent massive infarct. **Figure S3**. Acute ischemic encephalopathy in ARCD. **Figure S4**. Circle of Willis in ARCD. **Figure S5**. Small arteries in the deep white matter have thickened walls in ARCD. **Figure S6**. Periventricular leukoencephalopathy in ARCD. **Figure S7**. Extracerebral pathology in ARCD. **Figure S8**. Systemic involvement of large and small arteries in ARCD. (PDF 10665 kb)
