# Supplementary material for: The defining pathology of the new clinical and histopathologic entity ACTA2-related cerebrovascular disease
Source: Acta Neuropathol Commun. 2015 Dec 4;3:81. doi: 10.1186/s40478-015-0262-7 (PMC4670506; doi:10.1186/s40478-015-0262-7)
Supplement: Additional file 1: — Figure S1. CT angiography measurements of luminal cross sectional area and diameter in ARCD. Figure S2. Gross appearance of the ARCD brain with recent massive infarct. Figure S3. Acute ischemic encephalopathy in ARCD. Figure S4. Circle of Willis in ARCD. Figure S5. Small arteries in the deep white matter have thickened walls in ARCD. Figure S6. Periventricular leukoencephalopathy in ARCD. Figure S7. Extracerebral pathology in ARCD. Figure S8. Systemic involvement of large and small arteries in ARCD. (PDF 10665 kb) [file 40478_2015_262_MOESM1_ESM.pdf]

Additional file 1

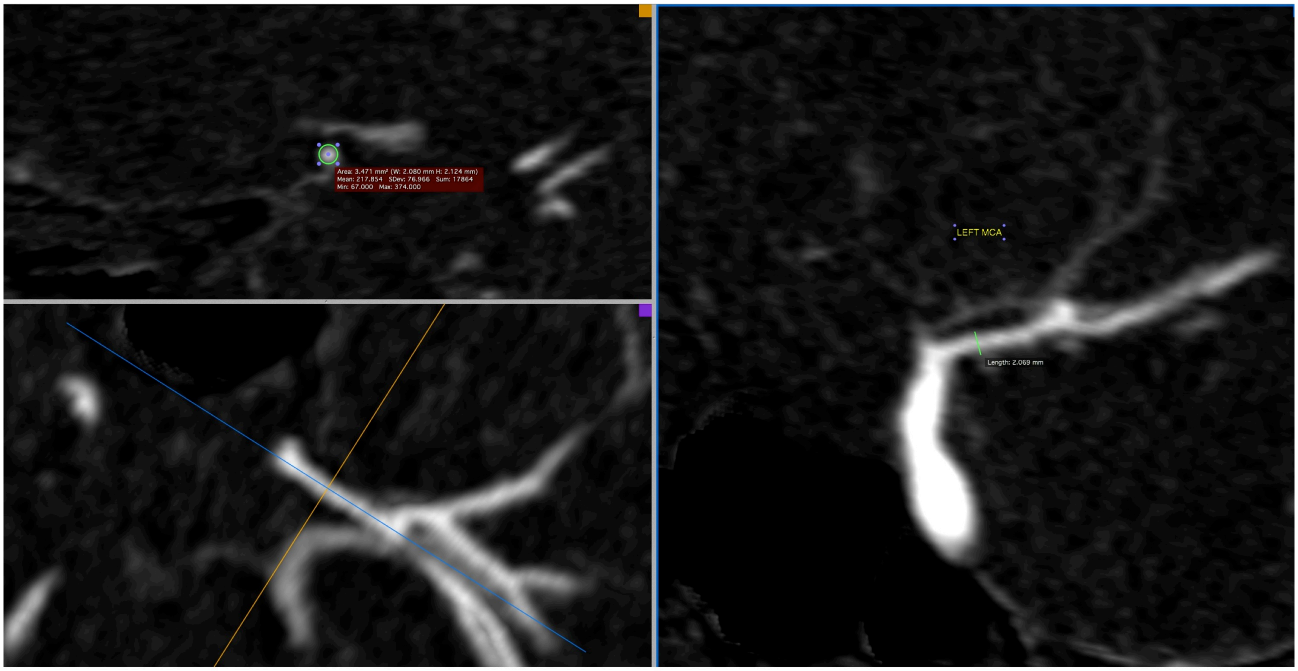

Figure S1. CT angiography measurements of luminal cross sectional area and diameter in ARCD. CT angiography source images of the large cerebral arteries were used and the measurements of the left MCA from the ARCD patient are shown. Circular measurements were obtained for cross sectional area in the short axis views (left images). Linear measurements were obtained for luminal diameter in the mid portion of the vessels on long axis views (right image).

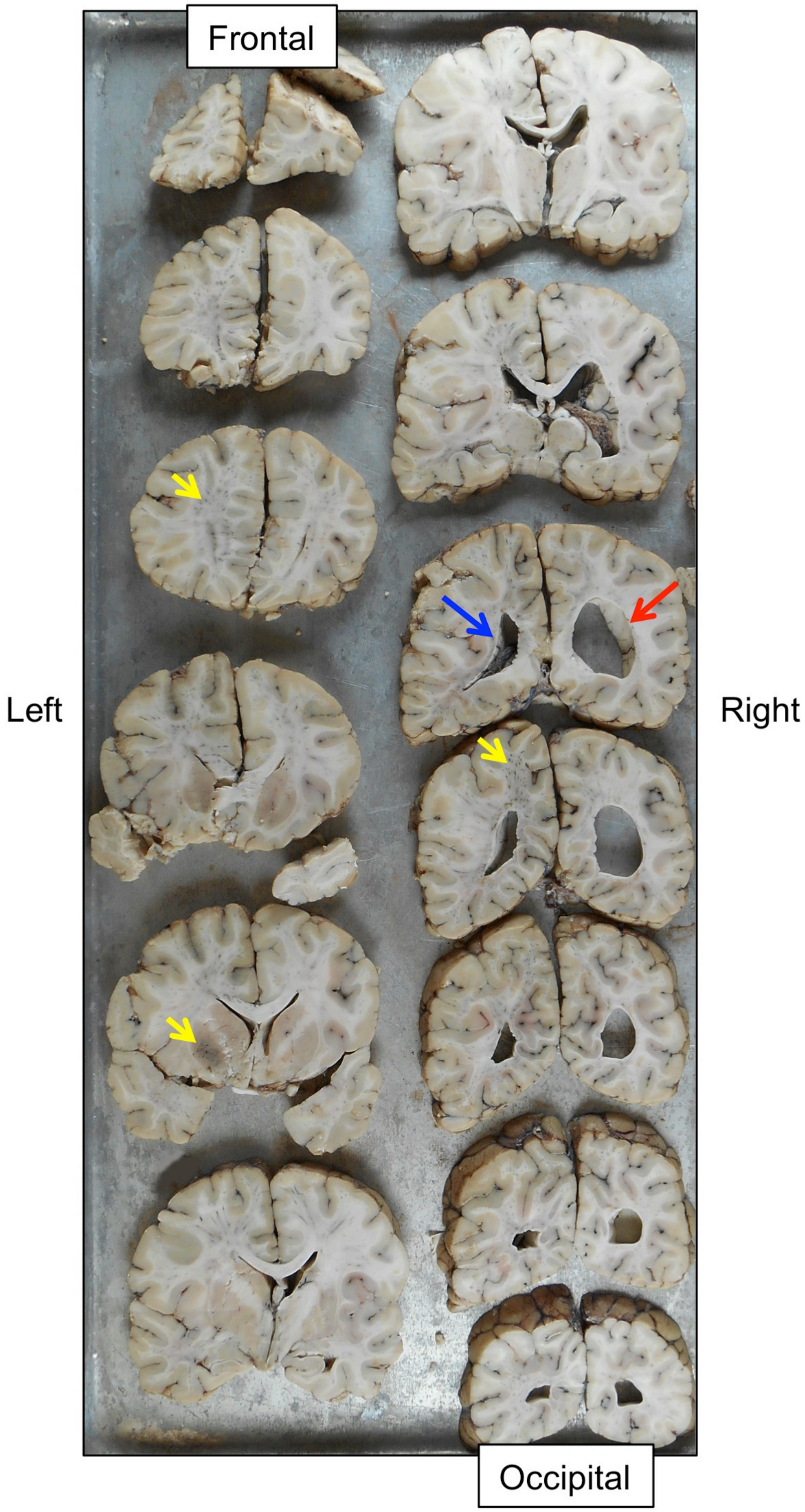

Figure S2. Gross appearance of the ARCD brain with recent massive infarct. Serial sections of cerebrum show signs of left cerebral edema with compression of the left lateral ventricle (blue arrow), mild midline shift (best seen in the 5th section), and prominence of dilated vessels in the left white matter and basal ganglia (yellow arrows). The colpocephaly-like dilatation of the lateral ventricles is best seen on the right (red arrow), in the absence of the compressing effect of edema.

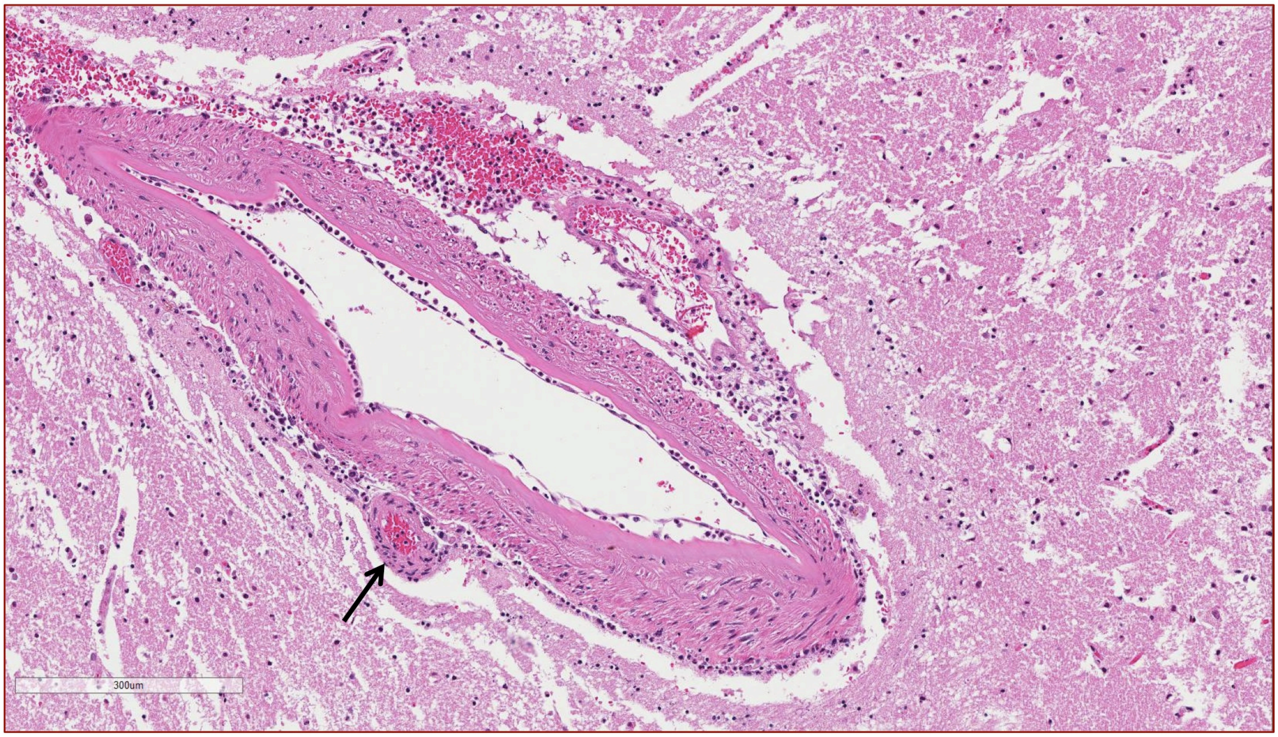

Figure S3. Acute ischemic encephalopathy in ARCD. H&E stained section through left basal ganglia shows margination of neutrophils in a mid-size artery, sparse neutrophilic infiltration of the neuropil and acute neuronal and glial necrosis. Note thickened walls of mid-size and small size (arrow) arteries.

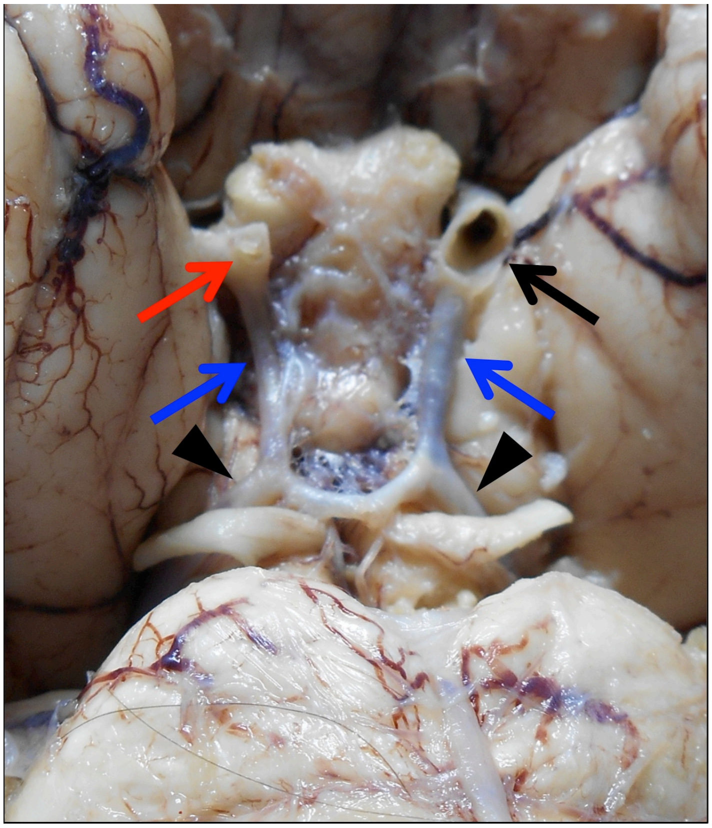

Figure S4. Circle of Willis in ARCD. View of the base of the brain showing the right ICA with severe narrowing of the lumen by intimal proliferation (red arrow), the left ICA (black arrow), well-developed posterior communicans arteries (blue arrows) and the PCAs (black arrowheads).

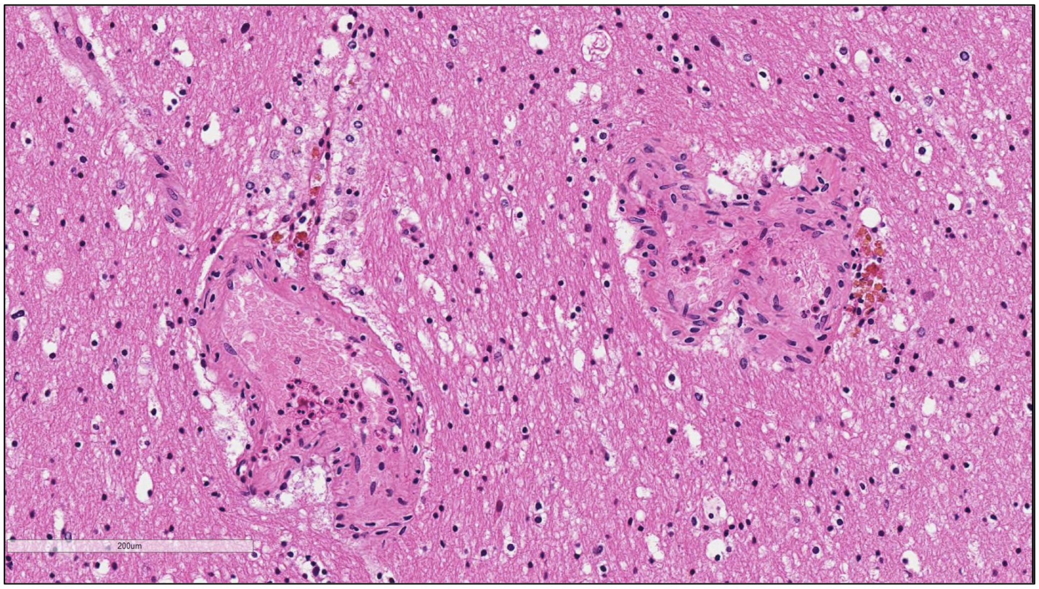

Figure S5. Small arteries in the deep white matter have thickened walls in ARCD. H&E section of left hemispheric deep white matter showing small size arteries.

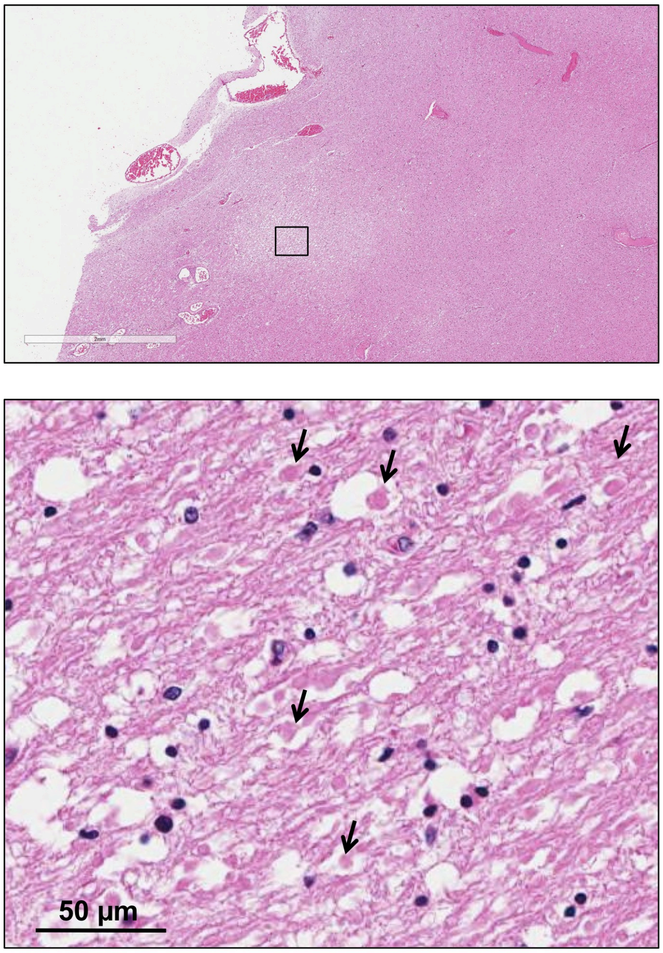

Figure S6 Periventricular leukoencephalopathy in ARCD. H&E sections of periventricular white matter show areas of rarefaction (upper panel). The square is magnified in the lower panel to illustrate numerous axonal spheroids (arrows).

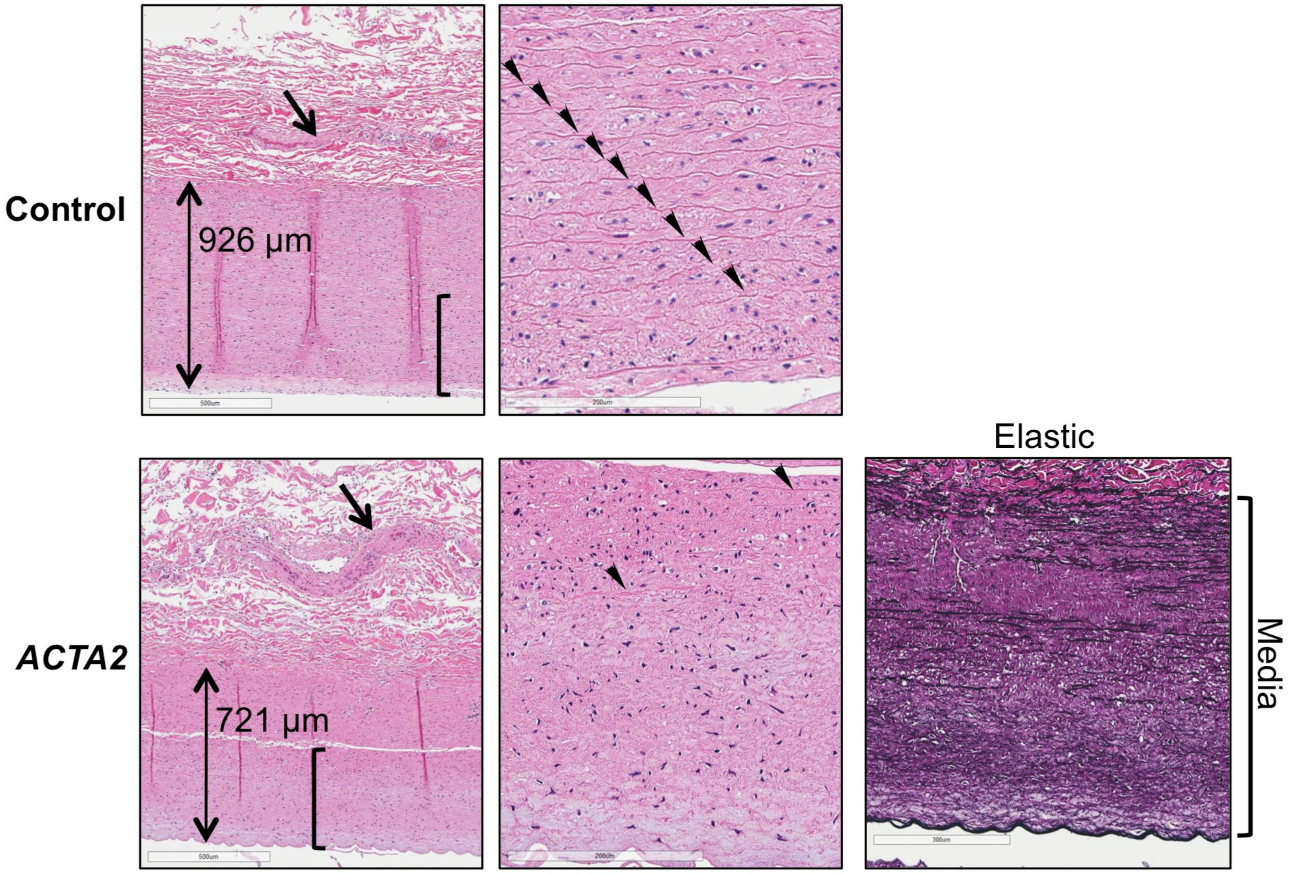

Figure S7. Extracerebral pathology in ARCD. H&E sections of aorta show thinning of the combined intima and media layers (double headed arrow), marked fragmentation and loss of the elastic lamellae (arrowheads in the magnified regions marked by brackets) and vasa vasorum with thickened walls (arrows) in the ARCD patient in comparison to the normal control. An elastic stain confirms the fragmentation and loss of the elastic lamellae in the ARCD patient.

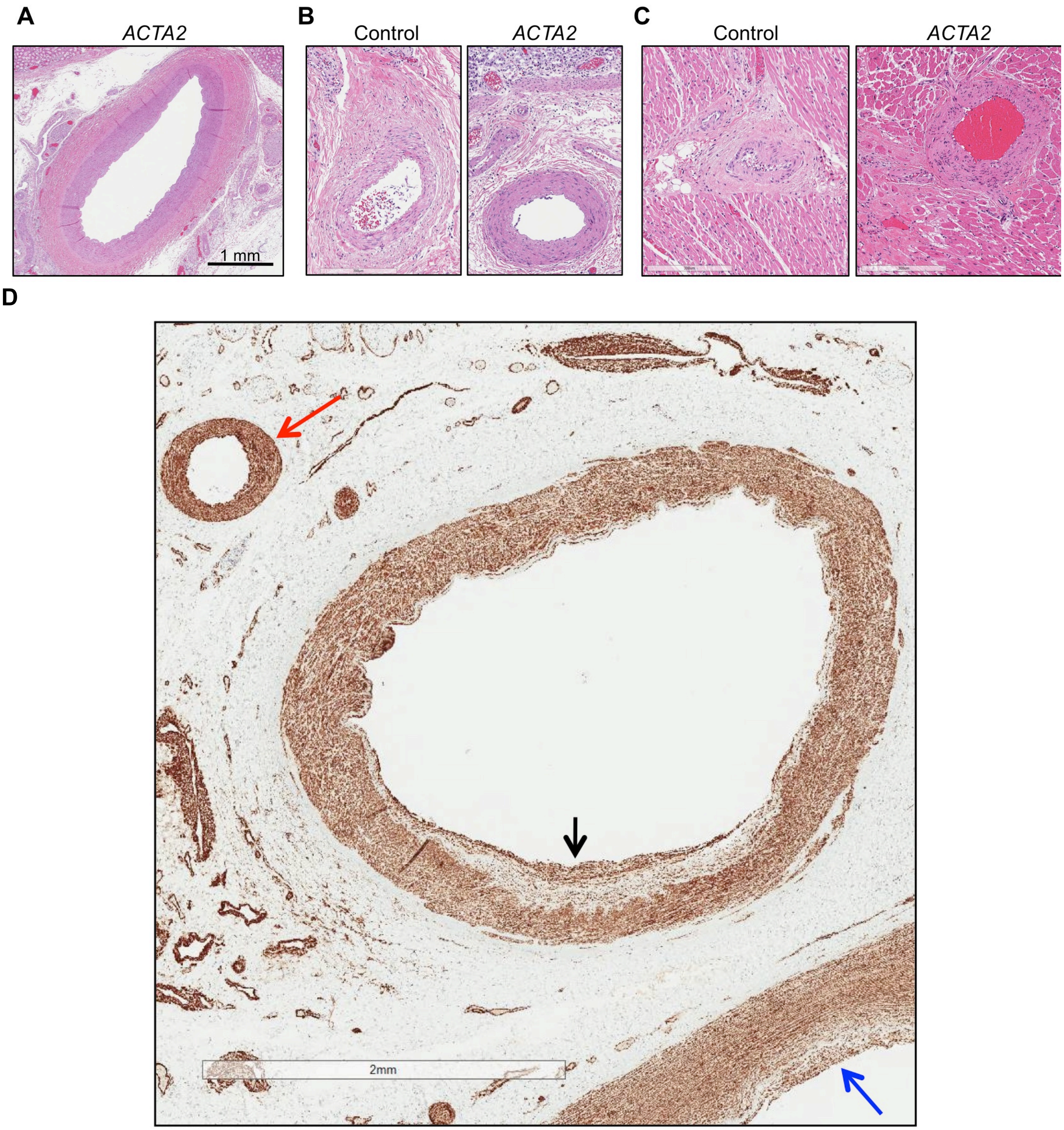

Figure S8. Systemic involvement of large and small arteries in ARCD. **A.** H&E section through renal hilum showing a segmental renal artery with subintimal SMC proliferation. Small arteries with Increased wall thickness are noted in the hilar fat. **B.** H&E sections of control and *ACTA2* mutant stomach submucosa show Increased wall thickness of small-size arteries in the *ACTA2* mutant. **C.** H&E sections of left ventricle wall show increased vessel wall thickness of small-size branches of the coronary artery in the *ACTA2* mutant. Note associated cardiomyocyte hypertrophy and interstitial fibrosis. **D.** IHC with anti-α-SMA antibody of a section of aorta (blue arrow) with an aortic branch showing intimal SMC proliferation (black arrow). A small vessel with SMC proliferation and thickened wall is indicated by the red arrow.
